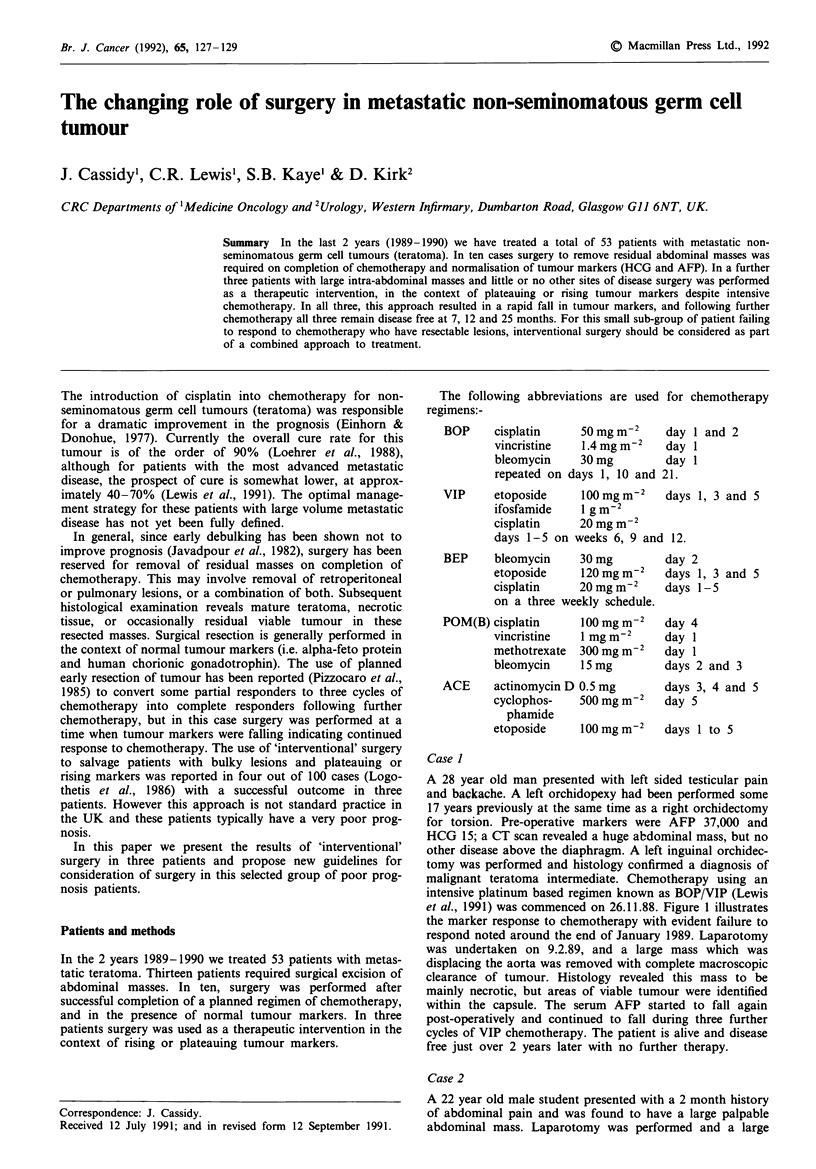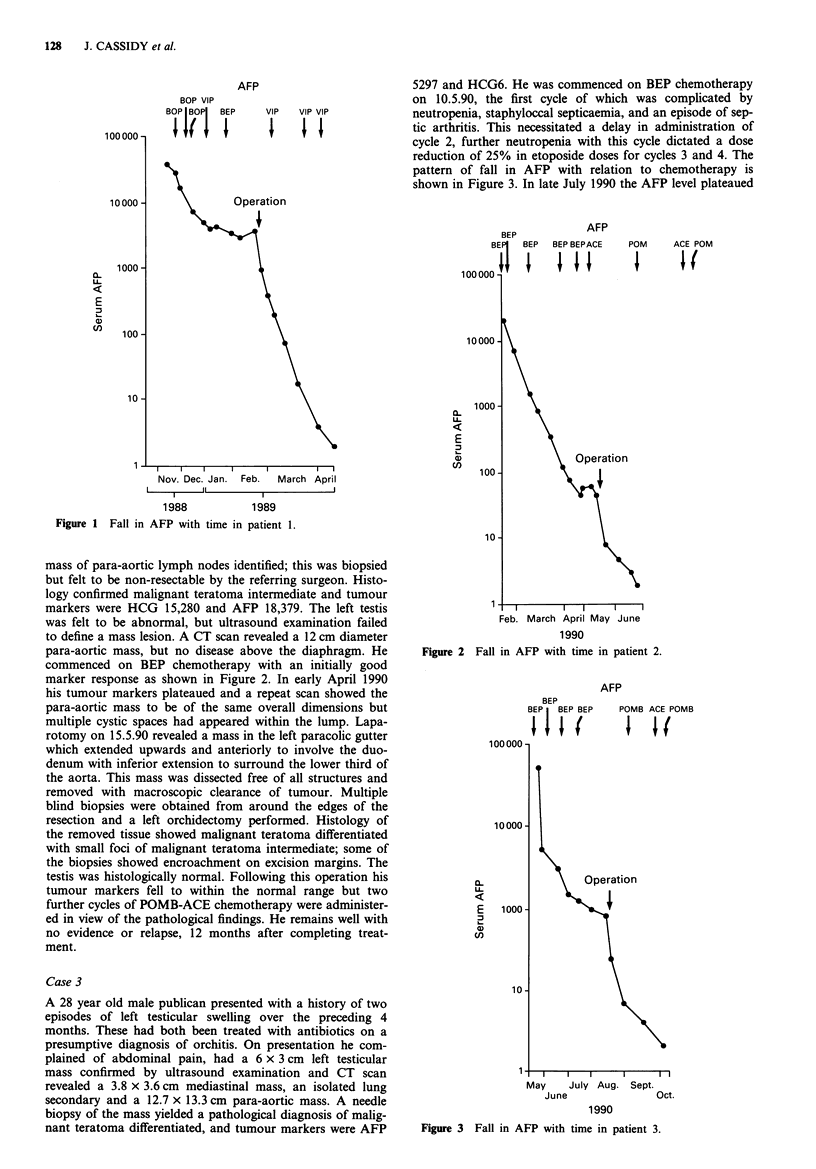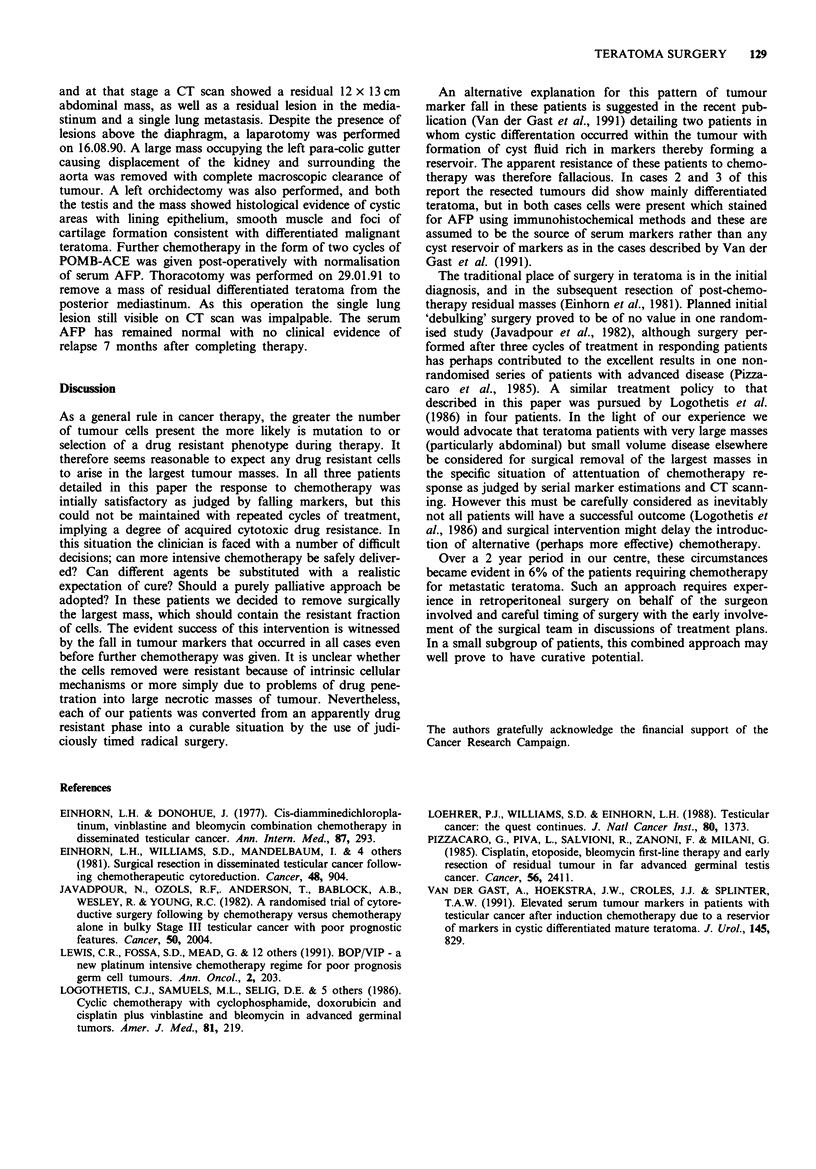# The changing role of surgery in metastatic non-seminomatous germ cell tumour.

**DOI:** 10.1038/bjc.1992.24

**Published:** 1992-01

**Authors:** J. Cassidy, C. R. Lewis, S. B. Kaye, D. Kirk

**Affiliations:** CRC Department of Medicine Oncology, Western Infirmary, Glasgow, UK.

## Abstract

In the last 2 years (1989-1990) we have treated a total of 53 patients with metastatic nonseminomatous germ cell tumours (teratoma). In ten cases surgery to remove residual abdominal masses was required on completion of chemotherapy and normalisation of tumour markers (HCG and AFP). In a further three patients with large intra-abdominal masses and little or no other sites of disease surgery was performed as a therapeutic intervention, in the context of plateauing or rising tumour markers despite intensive chemotherapy. In all three, this approach resulted in a rapid fall in tumour markers, and following further chemotherapy all three remain disease free at 7, 12 and 25 months. For this small sub-group of patient failing to respond to chemotherapy who have resectable lesions, interventional surgery should be considered as part of a combined approach to treatment.


*Br. J. Cancer* (1992), **65**, 127–129
© Macmillan Press Ltd., 1992

### The changing role of surgery in metastatic non-seminomatous germ cell tumour

J. Cassidy[1], C.R. Lewis[1], S.B. Kaye[1] & D. Kirk[2]

*CRC Departments of [1]Medicine Oncology and [2]Urology, Western Infirmary, Dumbarton Road, Glasgow G11 6NT, UK.*

**Summary** In the last 2 years (1989–1990) we have treated a total of 53 patients with metastatic non-seminomatous germ cell tumours (teratoma). In ten cases surgery to remove residual abdominal masses was required on completion of chemotherapy and normalisation of tumour markers (HCG and AFP). In a further three patients with large intra-abdominal masses and little or no other sites of disease surgery was performed as a therapeutic intervention, in the context of plateauing or rising tumour markers despite intensive chemotherapy. In all three, this approach resulted in a rapid fall in tumour markers, and following further chemotherapy all three remain disease free at 7, 12 and 25 months. For this small sub-group of patient failing to respond to chemotherapy who have resectable lesions, interventional surgery should be considered as part of a combined approach to treatment.

The introduction of cisplatin into chemotherapy for non-seminomatous germ cell tumours (teratoma) was responsible for a dramatic improvement in the prognosis (Einhorn & Donohue, 1977). Currently the overall cure rate for this tumour is of the order of 90% (Loehrer *et al.*, 1988), although for patients with the most advanced metastatic disease, the prospect of cure is somewhat lower, at approximately 40–70% (Lewis *et al.*, 1991). The optimal management strategy for these patients with large volume metastatic disease has not yet been fully defined.

In general, since early debulking has been shown not to improve prognosis (Javadpour *et al.*, 1982), surgery has been reserved for removal of residual masses on completion of chemotherapy. This may involve removal of retroperitoneal or pulmonary lesions, or a combination of both. Subsequent histological examination reveals mature teratoma, necrotic tissue, or occasionally residual viable tumour in these resected masses. Surgical resection is generally performed in the context of normal tumour markers (i.e. alpha-feto protein and human chorionic gonadotrophin). The use of planned early resection of tumour has been reported (Pizzocaro *et al.*, 1985) to convert some partial responders to three cycles of chemotherapy into complete responders following further chemotherapy, but in this case surgery was performed at a time when tumour markers were falling indicating continued response to chemotherapy. The use of 'interventional' surgery to salvage patients with bulky lesions and plateauing or rising markers was reported in four out of 100 cases (Logothetis *et al.*, 1986) with a successful outcome in three patients. However this approach is not standard practice in the UK and these patients typically have a very poor prognosis.

In this paper we present the results of 'interventional' surgery in three patients and propose new guidelines for consideration of surgery in this selected group of poor prognosis patients.

### Patients and methods

In the 2 years 1989–1990 we treated 53 patients with metastatic teratoma. Thirteen patients required surgical excision of abdominal masses. In ten, surgery was performed after successful completion of a planned regimen of chemotherapy, and in the presence of normal tumour markers. In three patients surgery was used as a therapeutic intervention in the context of rising or plateauing tumour markers.

Correspondence: J. Cassidy.
Received 12 July 1991; and in revised form 12 September 1991.

The following abbreviations are used for chemotherapy regimens:-

| | | | |
|---|---|---|---|
| BOP | cisplatin | 50 mg $m^{-2}$ | day 1 and 2 |
| | vincristine | 1.4 mg $m^{-2}$ | day 1 |
| | bleomycin | 30 mg | day 1 |
| | repeated on days 1, 10 and 21. | | |
| VIP | etoposide | 100 mg $m^{-2}$ | days 1, 3 and 5 |
| | ifosfamide | 1 g $m^{-2}$ | |
| | cisplatin | 20 mg $m^{-2}$ | |
| | days 1–5 on weeks 6, 9 and 12. | | |
| BEP | bleomycin | 30 mg | day 2 |
| | etoposide | 120 mg $m^{-2}$ | days 1, 3 and 5 |
| | cisplatin | 20 mg $m^{-2}$ | days 1–5 |
| | on a three weekly schedule. | | |
| POM(B) | cisplatin | 100 mg $m^{-2}$ | day 4 |
| | vincristine | 1 mg $m^{-2}$ | day 1 |
| | methotrexate | 300 mg $m^{-2}$ | day 1 |
| | bleomycin | 15 mg | days 2 and 3 |
| ACE | actinomycin D | 0.5 mg | days 3, 4 and 5 |
| | cyclophos-phamide | 500 mg $m^{-2}$ | day 5 |
| | etoposide | 100 mg $m^{-2}$ | days 1 to 5 |

*Case 1*

A 28 year old man presented with left sided testicular pain and backache. A left orchidopexy had been performed some 17 years previously at the same time as a right orchidectomy for torsion. Pre-operative markers were AFP 37,000 and HCG 15; a CT scan revealed a huge abdominal mass, but no other disease above the diaphragm. A left inguinal orchidectomy was performed and histology confirmed a diagnosis of malignant teratoma intermediate. Chemotherapy using an intensive platinum based regimen known as BOP/VIP (Lewis *et al.*, 1991) was commenced on 26.11.88. Figure 1 illustrates the marker response to chemotherapy with evident failure to respond noted around the end of January 1989. Laparotomy was undertaken on 9.2.89, and a large mass which was displacing the aorta was removed with complete macroscopic clearance of tumour. Histology revealed this mass to be mainly necrotic, but areas of viable tumour were identified within the capsule. The serum AFP started to fall again post-operatively and continued to fall during three further cycles of VIP chemotherapy. The patient is alive and disease free just over 2 years later with no further therapy.

*Case 2*

A 22 year old male student presented with a 2 month history of abdominal pain and was found to have a large palpable abdominal mass. Laparotomy was performed and a large

**Figure 1** Fall in AFP with time in patient 1.

mass of para-aortic lymph nodes identified; this was biopsied but felt to be non-resectable by the referring surgeon. Histology confirmed malignant teratoma intermediate and tumour markers were HCG 15,280 and AFP 18,379. The left testis was felt to be abnormal, but ultrasound examination failed to define a mass lesion. A CT scan revealed a 12 cm diameter para-aortic mass, but no disease above the diaphragm. He commenced on BEP chemotherapy with an initially good marker response as shown in Figure 2. In early April 1990 his tumour markers plateaued and a repeat scan showed the para-aortic mass to be of the same overall dimensions but multiple cystic spaces had appeared within the lump. Laparotomy on 15.5.90 revealed a mass in the left paracolic gutter which extended upwards and anteriorly to involve the duodenum with inferior extension to surround the lower third of the aorta. This mass was dissected free of all structures and removed with macroscopic clearance of tumour. Multiple blind biopsies were obtained from around the edges of the resection and a left orchidectomy performed. Histology of the removed tissue showed malignant teratoma differentiated with small foci of malignant teratoma intermediate; some of the biopsies showed encroachment on excision margins. The testis was histologically normal. Following this operation his tumour markers fell to within the normal range but two further cycles of POMB-ACE chemotherapy were administered in view of the pathological findings. He remains well with no evidence or relapse, 12 months after completing treatment.

*Case 3*

A 28 year old male publican presented with a history of two episodes of left testicular swelling over the preceding 4 months. These had both been treated with antibiotics on a presumptive diagnosis of orchitis. On presentation he complained of abdominal pain, had a 6 × 3 cm left testicular mass confirmed by ultrasound examination and CT scan revealed a 3.8 × 3.6 cm mediastinal mass, an isolated lung secondary and a 12.7 × 13.3 cm para-aortic mass. A needle biopsy of the mass yielded a pathological diagnosis of malignant teratoma differentiated, and tumour markers were AFP 5297 and HCG6. He was commenced on BEP chemotherapy on 10.5.90, the first cycle of which was complicated by neutropenia, staphyloccal septicaemia, and an episode of septic arthritis. This necessitated a delay in administration of cycle 2, further neutropenia with this cycle dictated a dose reduction of 25% in etoposide doses for cycles 3 and 4. The pattern of fall in AFP with relation to chemotherapy is shown in Figure 3. In late July 1990 the AFP level plateaued

**Figure 2** Fall in AFP with time in patient 2.

**Figure 3** Fall in AFP with time in patient 3.

and at that stage a CT scan showed a residual 12 × 13 cm abdominal mass, as well as a residual lesion in the mediastinum and a single lung metastasis. Despite the presence of lesions above the diaphragm, a laparotomy was performed on 16.08.90. A large mass occupying the left para-colic gutter causing displacement of the kidney and surrounding the aorta was removed with complete macroscopic clearance of tumour. A left orchidectomy was also performed, and both the testis and the mass showed histological evidence of cystic areas with lining epithelium, smooth muscle and foci of cartilage formation consistent with differentiated malignant teratoma. Further chemotherapy in the form of two cycles of POMB-ACE was given post-operatively with normalisation of serum AFP. Thoracotomy was performed on 29.01.91 to remove a mass of residual differentiated teratoma from the posterior mediastinum. As this operation the single lung lesion still visible on CT scan was impalpable. The serum AFP has remained normal with no clinical evidence of relapse 7 months after completing therapy.

### Discussion

As a general rule in cancer therapy, the greater the number of tumour cells present the more likely is mutation to or selection of a drug resistant phenotype during therapy. It therefore seems reasonable to expect any drug resistant cells to arise in the largest tumour masses. In all three patients detailed in this paper the response to chemotherapy was intially satisfactory as judged by falling markers, but this could not be maintained with repeated cycles of treatment, implying a degree of acquired cytotoxic drug resistance. In this situation the clinician is faced with a number of difficult decisions; can more intensive chemotherapy be safely delivered? Can different agents be substituted with a realistic expectation of cure? Should a purely palliative approach be adopted? In these patients we decided to remove surgically the largest mass, which should contain the resistant fraction of cells. The evident success of this intervention is witnessed by the fall in tumour markers that occurred in all cases even before further chemotherapy was given. It is unclear whether the cells removed were resistant because of intrinsic cellular mechanisms or more simply due to problems of drug penetration into large necrotic masses of tumour. Nevertheless, each of our patients was converted from an apparently drug resistant phase into a curable situation by the use of judiciously timed radical surgery.

An alternative explanation for this pattern of tumour marker fall in these patients is suggested in the recent publication (Van der Gast *et al.*, 1991) detailing two patients in whom cystic differentation occurred within the tumour with formation of cyst fluid rich in markers thereby forming a reservoir. The apparent resistance of these patients to chemotherapy was therefore fallacious. In cases 2 and 3 of this report the resected tumours did show mainly differentiated teratoma, but in both cases cells were present which stained for AFP using immunohistochemical methods and these are assumed to be the source of serum markers rather than any cyst reservoir of markers as in the cases described by Van der Gast *et al.* (1991).

The traditional place of surgery in teratoma is in the initial diagnosis, and in the subsequent resection of post-chemotherapy residual masses (Einhorn *et al.*, 1981). Planned initial 'debulking' surgery proved to be of no value in one randomised study (Javadpour *et al.*, 1982), although surgery performed after three cycles of treatment in responding patients has perhaps contributed to the excellent results in one non-randomised series of patients with advanced disease (Pizzacaro *et al.*, 1985). A similar treatment policy to that described in this paper was pursued by Logothetis *et al.* (1986) in four patients. In the light of our experience we would advocate that teratoma patients with very large masses (particularly abdominal) but small volume disease elsewhere be considered for surgical removal of the largest masses in the specific situation of attentuation of chemotherapy response as judged by serial marker estimations and CT scanning. However this must be carefully considered as inevitably not all patients will have a successful outcome (Logothetis *et al.*, 1986) and surgical intervention might delay the introduction of alternative (perhaps more effective) chemotherapy.

Over a 2 year period in our centre, these circumstances became evident in 6% of the patients requiring chemotherapy for metastatic teratoma. Such an approach requires experience in retroperitoneal surgery on behalf of the surgeon involved and careful timing of surgery with the early involvement of the surgical team in discussions of treatment plans. In a small subgroup of patients, this combined approach may well prove to have curative potential.

The authors gratefully acknowledge the financial support of the Cancer Research Campaign.

### References

EINHORN, L.H. & DONOHUE, J. (1977). Cis-diamminedichloroplatinum, vinblastine and bleomycin combination chemotherapy in disseminated testicular cancer. *Ann. Intern. Med.*, **87**, 293.

EINHORN, L.H., WILLIAMS, S.D., MANDELBAUM, I. & 4 others (1981). Surgical resection in disseminated testicular cancer following chemotherapeutic cytoreduction. *Cancer*, **48**, 904.

JAVADPOUR, N., OZOLS, R.F,. ANDERSON, T., BABLOCK, A.B., WESLEY, R. & YOUNG, R.C. (1982). A randomised trial of cytoreductive surgery following by chemotherapy versus chemotherapy alone in bulky Stage III testicular cancer with poor prognostic features. *Cancer*, **50**, 2004.

LEWIS, C.R., FOSSA, S.D., MEAD, G. & 12 others (1991). BOP/VIP - a new platinum intensive chemotherapy regime for poor prognosis germ cell tumours. *Ann. Oncol.*, **2**, 203.

LOGOTHETIS, C.J., SAMUELS, M.L., SELIG, D.E. & 5 others (1986). Cyclic chemotherapy with cyclophosphamide, doxorubicin and cisplatin plus vinblastine and bleomycin in advanced germinal tumors. *Amer. J. Med.*, **81**, 219.

LOEHRER, P.J., WILLIAMS, S.D. & EINHORN, L.H. (1988). Testicular cancer: the quest continues. *J. Natl Cancer Inst.*, **80**, 1373.

PIZZACARO, G., PIVA, L., SALVIONI, R., ZANONI, F. & MILANI, G. (1985). Cisplatin, etoposide, bleomycin first-line therapy and early resection of residual tumour in far advanced germinal testis cancer. *Cancer*, **56**, 2411.

VAN DER GAST, A., HOEKSTRA, J.W., CROLES, J.J. & SPLINTER, T.A.W. (1991). Elevated serum tumour markers in patients with testicular cancer after induction chemotherapy due to a reservior of markers in cystic differentiated mature teratoma. *J. Urol.*, **145**, 829.